# Cross-sectional association between soda consumption and body mass index in a community-based sample of twins

**DOI:** 10.1186/s12937-017-0269-y

**Published:** 2017-08-22

**Authors:** Anna E. Eney, Siny Tsang, Joseph A. Delaney, Eric Turkheimer, Glen E. Duncan

**Affiliations:** 1 Nutritional Sciences Program, University of Washington, Seattle, WA 98195-3410 USA; 2 Department of Epidemiology, Columbia University, New York, NY 10032-2697 USA; 3 Department of Epidemiology, University of Washington, Seattle, WA 98195-7236 USA; 4 Department of Psychology, University of Virginia, Charlottesville, VA 22904-4400 USA; 5 Department of Nutrition and Exercise Physiology, Washington State University – Health Sciences Spokane, Box 1495, Spokane, WA 99210-1495 USA

**Keywords:** Nutrition, Twin registry, Public health, Body mass index

## Abstract

**Background:**

Consumption of sugar-sweetened beverages, such as soda, have been shown to play an important role in weight gain. Although soda consumption has been associated with body mass index (BMI) in many studies, it has been difficult to ascertain a true causal relationship between soda consumption and BMI for two reasons. First, findings have been based largely on observational and cross-sectional studies, with much less evidence from randomized controlled trials. Second, the reported relationships may be confounded by genetic and shared environmental factors that affect both soda consumption and BMI. In the present study, we used the twin design to better understand the relationship between soda consumption and BMI by accounting for measured and unmeasured confounds in non-experimental data. Associations from genetically informed tests in twins are considered “quasi-causal,” suggesting that our confidence in the causal underpinning of the association between soda consumption and BMI has been strengthened. We hypothesized that the association between soda consumption and BMI would be significant both between and within twins.

**Methods:**

This was a cross sectional study of 5787 same sex adult twin pairs (18–97 years, 66% female) from the community based Washington State Twin Registry. Structural equation modeling (SEM) was employed to investigate associations between soda consumption and BMI in the population (the phenotypic association between exposure and outcome among all twins treated as individuals) and within pairs of identical and fraternal twins (the quasi-causal association controlling for between pair genetic and environmental confounds).

**Results:**

Among all twins, there was a significant phenotypic association between soda consumption and BMI that held when controlling for age, sex, race, annual household income, and education level (*P* < 0.05). In the quasi-causal model, however, the effect of soda consumption on BMI was greatly reduced and no longer significant, with a large genetic confound in both men and women (*P* < 0.05).

**Conclusion:**

Among a large group of adult twin pairs, increased soda consumption was associated with increased BMI; however, the observed association was mediated by a genetic background common to both.

## Background

The prevalence of obesity has more than doubled in the past 30 years in the U.S., with over one-third of adults currently categorized as obese based on body mass index (BMI) [1]. The high prevalence of obesity in the U.S. population has raised public health concern because it is associated with chronic diseases such as cardiovascular disease, type 2 diabetes, and some forms of cancer [2–5]. In turn, chronic diseases are the leading cause of poor health, disability, and death, and account for most of health-care expenditures, among the U.S. population [6]. In order to improve population health, it is imperative to gain a better understanding of factors affecting the development of obesity.

Obesity is complex and its development is influenced by multiple factors ranging from biology to policy [7]. With respect to biologic factors, many studies have shown strong genetic and epigenetic determinants to weight regulation systems and BMI [8–14]. In addition, the complex interplay between genetic and environmental factors in obesity are well documented [13, 15, 16]. Genetic and shared environmental factors have also been implicated in food preferences [17–19], including the consumption of sweet-tasting carbohydrate sources [20, 21]. This suggests that dietary behavior, an important lifestyle factor influencing obesity, has some underlying influence from shared familial factors along genetic and environmental lines.

Among the many dietary influences on obesity, consumption of sugar-sweetened beverages (SSBs) such as soda have been shown to play an important role in weight gain. A recent systematic review and meta-analysis of prospective cohort studies (15 in children and 7 in adults) and randomized controlled trials (5 each in children and adults) provides evidence that SSB consumption is associated with weight gain in children and adults [22]. Interestingly, a recent report found evidence that experimental studies that have financial conflicts with the SSB industry are more likely than independently funded ones to find no relationship between SSB consumption and metabolic outcomes, including obesity [23], thus contributing to an ongoing debate surrounding *causal* links between SSBs and health outcomes [24, 25].

Although there is compelling data suggesting that SSB consumption, including soda, is *associated* with adiposity measures including higher BMI, the reported relationships may also be confounded by genetic and shared environmental factors that affect both soda drinking and BMI. It might be, for example, that genetic predispositions to soda drinking also have an effect on BMI, inducing a statistical association in the absence of a causal effect. Similarly, shared environmental variables such as parental dietary pattern, parent food modeling, and socioeconomic status could affect both child food preferences and BMI [26], once again inducing a correlation in the absence of a causal effect. More specifically, child soda consumption and the soda consumption patterns of parents and friends are highly inter-related, suggesting that a child’s rearing environment plays an important role in a child’s consumptionof soda [27]. Other factors associated with youth soda consumption include innate taste preferences and access to soda in the home and school [27].

Therefore, to gain a better understanding of the association between BMI and soda consumption, genetic and shared environmental factors must be adequately controlled. Twin designs are a powerful tool for understanding genetic, shared environmental, and non-shared environmental factors and their effects on a range of human traits [28]. This study design provides further insight into the documented association between soda consumption and BMI by determining whether or not the association is confounded by genetic and shared environmental factors between exposure and outcome, indicative of a “quasi-causal” relationship [29]. We hypothesized that the association between soda consumption and BMI would be significant both between and within twins.

## Methods

### Subjects

This secondary data analysis included a sample of 5787 twin pairs from the community-based Washington State Twin Registry within a cross-sectional study design. Twins include both monozygotic (MZ) and dizygotic (DZ) male and female twin pairs of the same sex, aged 18–97 years, reared together. Participants were recruited from Washington State driver’s license and identification card applications [30]. All twins completed an enrollment survey with questions related to childhood similarity to evaluate twin zygosity (MZ vs. DZ), a common twin registry practice with an accuracy of 95–98% compared to biological indicators [31, 32]. Twins were mailed an invitation letter and enrollment survey including questions related to height, weight, and soda consumption. Data collected from completed questionnaires received between 2009 and 2015 were analyzed.

### Measures

Body Mass Index. The main outcome, BMI, was calculated from self-reported height and weight and expressed as kg/m^2^. The height and weight measures were collected from responses to the survey questions “What is your current height?” in feet and inches and “What is your current weight?” in pounds.

Soda Consumption. The predictor variable was soda consumption, which was collected from self-reported dietary recall based on the question “During the past 4 weeks, how many servings of the following did you have on a typical day…Cans or glasses of soda?” Possible answers included “none”, “1–2”, “3–4”, or “5 + .” Because many twins are initially recruited into the Registry at age 18, this question was taken from the Youth Risk Behavior Surveillance System (YRBSS). Methodology of the YRBSS is described elsewhere [33]. The YRBSS soda question has been evaluated previously; Park et al. [34] report unpublished data demonstrating a significant correlation (*r* = 0.44) between soda intake from YRBSS and a 24-h dietary recall among high school students, whereas O’Malley et al. [35] report that mean intakes of soda from YRBSS and three, 24-h dietary recalls were not significantly different from each other as well as a significant corrected Pearson’s correlation between methods (*r* = 0.44; *p* < 0.001) among 615 high school students.

Covariates. Age, sex, race, annual household income, and education level were collected from responses to survey questions and used as covariates in the statistical analyses. Age at time of survey was calculated based on reported date of birth. Sex was reported as male or female. Race was reported using six standard response options (American Indian or Alaska Native, Black or African-American, Native Hawaiian or Pacific Islander, Asian, White, and Other), which was subsequently re-categorized as white and non-white. There were eight categories of income with the lowest being “less than $20,000” followed by “$20,000–29,999”, “$30,000–39,999”, and so on, ending with the highest category of “$80,000 or more”. Education (highest level of education completed) included five categories: grade 1–11, high school graduate/GED, some college, bachelor’s degree, and graduate/professional degree.

### Statistical analysis

BMI data were missing for 141 participants (1.2%), and soda consumption was missing for 95 (0.8%). These observations were omitted from descriptive analyses, but were included in the structural equation modeling analyses using full information maximum likelihood to account for missingness. In addition, 268 participants were missing zygosity information, and were therefore omitted from twin analyses. BMI was expressed as a continuous variable in all statistical analyses. In the structural equation analyses, soda drinking was modeled using a categorical variable model that posits a normally distributed latent continuous liability to soda consumption; latent cutoffs on the distribution determine placement of participants in the four measured categories [36].

**Fig. 1:**
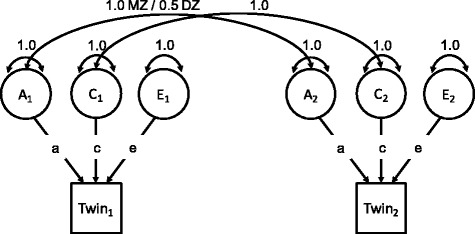
Univariate twin model. *A* additive genetic component; *C* shared environment component; *E* non-shared environment component

Descriptive statistics for subjects were computed and reported for the overall subject sample. Next, we used structural equation modeling [37] to fit a classical twin model to soda consumption and BMI (Fig. 1). The classical twin model uses the variances and co-variances of MZ and DZ twins to partition the variance of phenotypes into three components: the additive effect of genes (A), the environmental effect of being raised in the same family (C), and environmental effects that make siblings raised together different from each other (E). The latter term includes measurement error.

Partitioning of variability using the classical twin model was not the main goal of our analysis, however. Instead, our goal was to use the twin design to investigate the relationship between soda consumption and BMI between and within pairs of twins. In the absence of random assignment to soda-consumption conditions, an investigator cannot be certain that an observed association between soda consumption and BMI is actually the result of a causal effect. Phenotypic associations of this kind may also occur because genetic predispositions that lead to soda consumption are also associated with higher BMI, or alternatively because shared environmental background (e.g., poverty) predisposes to both soda consumption and high BMI.

Twin designs are especially useful for understanding measured and unmeasured uncontrolled confounds in non-experimental data. If the effect of soda consumption on BMI is truly causal, then one would expect it to be manifest both between twin pairs (pairs consuming more soda on average would have higher average BMI) and within pairs (the member of a pair who consumes more soda would have higher BMI than the co-twin who drinks less). If, however, the association is the result of uncontrolled confounding variables such as genetic background or socioeconomic status, the association will be observed between pairs but not within them, because twin pairs share a rearing environment and either all or half of their genetic background. The twin method cannot fully control for all potential confounds, however, and some uncontrolled variables may vary within pairs as well as between them. We therefore refer to associations that have survived genetically informed tests as “quasi-causal,” to suggest that the twin analysis has strengthened our confidence in the causal underpinning of the association.

**Fig. 2:**
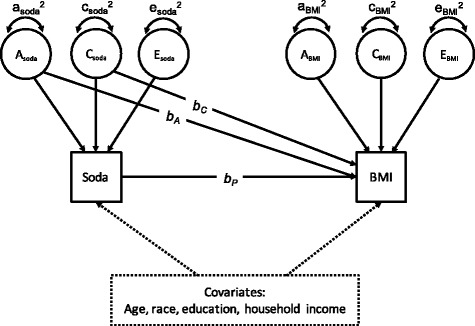
Quasi-causal twin model, controlling for covariates. *A* additive genetic component; *C* shared environment component; *E* non-shared environment component; *b*_*A*_ and *b*_*C*_ amount of residual variance of body mass index attributable to the genetic and shared environment, respectively; *b*_*P*_ phenotypic association

The logic of the method and the statistical methods associated with it are described in Turkheimer & Harden [29], and illustrated in Fig. 2. Soda consumption and BMI are both partitioned into ACE components using the classical twin method. In addition, BMI is regressed on phenotypic soda drinking (b_P_), as well as on the shared components (b_A_ and b_C_) of soda drinking. In the first analysis b_A_ and b_C_ are set to zero, leaving a simple regression of BMI on soda conumption at the individual level; this model is called a phenotypic association model, and tests for the association of soda consumption with BMI without including genetic or shared environmental confounds, but with other covariates as noted in the list above. The model is then re-estimated including estimates of b_A_ and b_C_, which controls for genetic and shared environmental confounds, respectively, in the estimation of the phenotypic effect. This is referred to as a quasi-causal model. The models were estimated first without and then with the set of covariates listed above.

All models were fit in Mplus 7.4 [37] using weighted least squares estimation. The alpha level for testing hypotheses was set to 0.05. Twin-based regression models are generally saturated, so the only source of reduced fit involves incidental issues such as differences between twins arbitrarily assigned as Twin 1 and Twin 2 within pairs. All reported models fit the data closely using standard “goodness of fit” tests.

## Results

### Descriptive statistics

Sample characteristics are provided in Table 1, overall and stratified by sex. Among all subjects, the average age was 43 yrs., 66% were female, mean BMI was 26.0 kg/m^2^, 37% reported an annual household income of 80 K+ per year, 80% had some college education, a bachelor’s degree or a graduate degree, and 92% of reported their race as white.

**Table 1:** Demographic characteristics of same sex twin pairs from the Washington State Twin Registry, 2009–2015

	Total	Men	Women
	(*n* = 5787)	(*n* = 1988)	(*n* = 3799)
Age	42.7 (17.9)	43.2 (18.9)	42.5 (17.4)
BMI (kg/m^2^)	26.0 (5.7)	26.3 (4.6)	25.9 (6.2)
Race (% White)	91.7	95.7	89.4
Household income (%)			
< 20 k	13.5	11.8	14.4
20 k – 29,999 k	8.4	8.1	8.6
30 k – 39,999 k	8.9	8.0	9.4
40 k – 49,999 k	8.4	7.3	9.0
50 k – 59,999 k	7.0	7.7	8.0
60 k – 69,999 k	7.5	7.3	7.7
70 k – 79,999 k	7.2	6.9	7.3
80 k+	37.2	42.8	35.7
Education (%)			
Less than high school	3.2	4.3	2.6
High school/GED	16.0	17.4	15.3
Some college	34.9	31.9	36.5
Bachelor’s degree	26.4	25.9	26.6
Graduate/professional degree	19.6	20.6	19.0
Soda consumption per day (%)			
No soda	60.0	55.6	62.4
1–2 sodas	29.4	31.6	28.2
3–4 sodas	6.3	7.6	5.6
5+ sodas	4.3	5.3	3.8

Continuous variables presented as mean (standard deviation) and categorical variables presented as percentages

### Univariate twin models

Table 2 shows the results of the univariate twin models of BMI and soda consumption. For BMI, in both males and females, the majority of the variance was attributable to additive genetics (Males = 63%, Females = 70%) with a small and non-significant proportion attributable to the shared environment (Males = 8%, Females = 6%) and the remainder attributable to the non-shared environment (Males = 29%, Females = 24%). The results were similar for soda consumption with 50% of the variance attributable to genetics in both males and females. In males, the shared environmental component estimated at negative and was set to zero; in females, the shared environmental component was positive, but small and non-significant.

**Table 2:** Twin intraclass correlations and standardized variance components for body mass index and soda consumption

	BMI (kg/m^2^)		Soda Consumption (servings per day)	
Twin correlations	Male	Female	Male	Female
*MZ*	0.71 (0.01)	0.76 (0.01)	0.50 (0.04)	0.56 (0.03)
*DZ*	0.40 (0.03)	0.41 (0.02)	0.25 (0.02)	0.31 (0.04)
ACE Estimates				
*a* ^*2*^	0.63 (0.07)	0.70 (0.04)	0.50 (0.03)	0.50 (0.09)
*c* ^*2*^	0.08 (0.07)	0.06 (0.04)	0.00 (0.00)^a^	0.06 (0.08)
*e* ^*2*^	0.29 (0.01)	0.24 (0.01)	0.50 (0.03)	0.44 (0.02)

Standard errors are presented within parentheses

*BMI* body mass index; *MZ* Monozygotic; *DZ* Dizygotic; *ACE* additive genetic, common environment, and unique environment variance components. ACE estimates are standardized biometric variance components obtained from the classical twin model decomposing the variance of BMI or soda consumption into additive genetic (A) variance, common environment (C) variance, and unique environment (E) variance

^a^The shared environmental component estimated at negative and was set to zero

### Phenotypic and quasi-causal analysis

Table 3 shows the results of the phenotypic (model 1) and quasi-causal models (2 and 3) without covariates. In the phenotypic model, there were significant effects of soda consumption on BMI in both males and females. Soda consumption accounted for 2.3% of the variability in BMI in males and 6.2% in females. In the quasi-causal model controlling for between pair genetic and environmental confounds, however, the effect of soda consumption on BMI was greatly reduced and no longer significant. The C confound had to be set to zero because there was no significant shared environmental variability in either males or females; with C set to zero there was a large genetic confound in both sexes, suggesting that the observed association between soda consumption and BMI was mediated by a genetic background common to both (*b*_*A*_, the amount of variance in body mass index attributable to additive genetic influences). The models could be fit to be equivalent in males and females without significant loss of fit (Table 3, model 3).

**Table 3:** Unstandardized parameter estimates estimating body mass index from soda consumption among same sex twins

	Model 1		Model 2		Model 3^a^	
	Phenotypic model		Quasi-causal model		Quasi-causal model	
	Male	Female	Male	Female	Male	Female
*b*_*A*_			**0.80 (0.31)**	**2.18 (0.27)**	**0.82 (0.24)**	**2.13 (0.23)**
*b*_*P*_	**0.70 (0.10)**	**1.53 (0.10)**	0.22 (0.14)	0.16 (0.13)	0.20 (0.10)	0.20 (0.10)
Goodness of fit						
RMSEA [90% CI]	0.03 [0.02, 0.04]		0.02 [0.01, 0.03]		0.02 [0.01, 0.03]	
CFI	0.988		0.996		0.996	
TLI	0.990		0.996		0.996	

Standard errors are presented within parentheses. The phenotypic model does not include controls for between-pair confounds, whereas quasi-causal models include controls for between-pair confounds. Bolded parameter estimates are statistically significant at *p* < 0.05

*b*_*A*_ amount of variance in body mass index attributable to additive genetic influences; *b*_*P*_ phenotypic association between predictor and outcome; *RMSEA* root mean square error of approximation; *CFI* comparative fit index; *TLI* Tucker-Lewis index

^a^ *b*_*P*_ is constrained to be equal for males and females

Results were very similar in the models including age, race, income and education as covariates (Table 4). In the phenotypic association model, there was once again a significant effect of soda consumption on BMI in both males and females. Age, race, income and education were all significant covariates. In the quasi-causal model, as above in the case of results with no covariates, the phenotypic effects were diminished and non-significant, with a substantial genetic confound in both males and females. The models could be fit to be equivalent in males and females without significant loss of fit (Table 4, model 3).

**Table 4:** Unstandardized parameter estimates estimating body mass index from soda consumption among same sex twins, with covariates

	Model 1		Model 2		Model 3^a^	
	Phenotypic model		Quasi-causal model		Quasi-causal model	
	Male	Female	Male	Female	Male	Female
*b*_*A*_			**1.22 (0.31)**	**2.16 (0.28)**	**1.28 (0.25)**	**2.06 (0.23)**
*b*_*P*_	**0.81 (0.09)**	**1.35 (0.10)**	0.14 (0.14)	0.03 (0.13)	0.10 (0.10)	0.10 (0.10)
Covariates						
Age	**0.79 (0.04)**	**0.71 (0.05)**	**0.78 (0.04)**	**0.68 (0.05)**	**0.77 (0.04)**	**0.68 (0.05)**
Race (White)	0.46 (0.32)	**−0.56 (0.27)**	0.37 (0.35)	**−0.63 (0.28)**	0.36 (0.32)	**−0.63 (0.28)**
Income	**0.10 (0.03)**	**−0.24 (0.04)**	**0.08 (0.04)**	**−0.29 (0.04)**	**0.07 (0.04)**	**−0.29 (0.04)**
Education	**−0.52 (0.13)**	**−0.74 (0.12)**	**−0.70 (0.14)**	**−1.03 (0.13)**	**−0.71 (0.13)**	**−1.02 (0.13)**
Goodness of fit						
RMSEA [90% CI]	0.03 [.020, .030]		0.02 [0.01, 0.03]		.02 [0.01, 0.03]	
CFI	0.984		0.990		0.990	
TLI	0.973		0.983		0.983	

Standard errors are presented within parentheses. The phenotypic model does not include controls for between-pair confounds, whereas quasi-causal models include controls for between-pair confounds. Bolded parameter estimates are statistically significant at *p* < 0.05

*b*_*A*_ amount of variance in body mass index attributable to additive genetic influences; *b*_*P*_ phenotypic association between predictor and outcome; *RMSEA* root mean square error of approximation; *CFI* comparative fit index; *TLI* Tucker-Lewis index

^a^ *b*_*P*_ is constrained to be equal for males and females

**Fig. 3:**
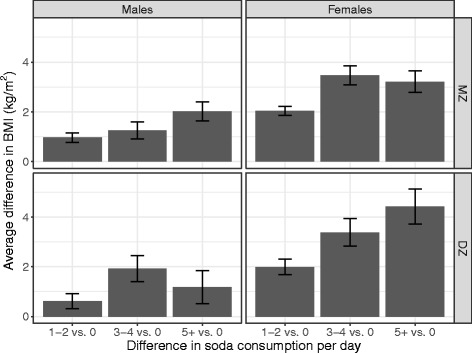
Difference in mean body mass index between participants consuming no soda and the three levels of soda consumption

**Fig. 4:**
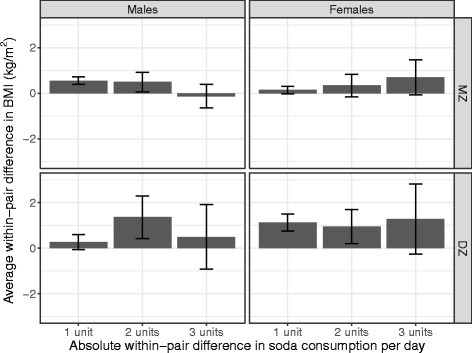
Difference in body mass index between member of the pair consuming more soda and member consuming less

Figures 3 and 4 illustrate the differences between the significant phenotypic effect and the non-significant quasi-causal effect, respectively. Note that these figures merely illustrate the effect (or lack thereof) demonstrated in the model; the model is implicitly based on within and between pair variances but no between and within pair difference scores are computed. Figure 3 shows the difference in mean BMI between participants consuming no soda and the three levels of soda consumption; for males and females and MZ and DZ pairs, mean BMI increases with increasing levels of soda consumption. Figure 4, in contrast, illustrates the within pair difference in BMI between the member of the pair consuming more soda and the member of the pair consuming less soda, broken down by the magnitude of the difference (one, two or three units of soda consumption; pairs consuming the same amount of soda were not included). There is no visible effect of soda consumption within pairs.

We performed sensitivity analyses by running all models excluding missing data. The results were fundamentally identical to those reported above, with two minor exceptions; the parameter estimates for income were no longer significant in models 2 and 3 for male twins, as previously found in Table 4 when analyses were run using full information maximum likelihood to account for missingness.

## Discussion

Among a large group of male and female twin pairs, soda consumption and BMI were significantly associated, with and without consideration of a set of common covariates. This finding is consistent with a preponderance of evidence demonstrating associations between SSBs, such as soda, and obesity-related measures, including BMI. However, as noted previously, the data supporting such findings is largely observational in nature, precluding causal inferences. In contrast, the major new finding of the present study is that the soda-BMI association was greatly reduced and no longer significant within twin pairs. The lack of association between soda and BMI within pairs was due to a large genetic confound between the exposure and outcome variables in both men and women, demonstrating that the observed association among all pairs was mediated by genetic factors that are common to both soda consumption and BMI.

The genetic factors that are common to both soda consumption and BMI are particularly strong among women, as evidenced by the large difference in parameter estimates between males and females in the quasi-causal models shown in Table 3. The male-female difference was attenuated but still present after covariate adjustment in Table 4. The commonly reported effect of soda consumption on BMI is illustrated in Fig. 3, showing increasing average differences in BMI as a function of increasing soda consumption of a magnitude that would imply biologic significance at the extremes (e.g., roughly 4.5 unit BMI difference in DZ females with 5+ vs. 0 sodas per day). When accounting for genetic and shared environmental confounds, however, the average within-pair difference in BMI is small and highly variable, regardless of the within-pair difference in soda consumption (Fig. 4).

The results of the present study demonstrate that the association between soda consumption and BMI should be examined within the context of genetic confounding. This suggestion is supported by the literature; here, we focus on studies that have examined SSBs and weight related outcomes while also considering genetic factors. A previous twin study reported that both diet and several anthropometric measures, including BMI, are influenced by genetic variation [21]. Interestingly, intrapair differences in the intake of sugar-sweetened soft drinks were associated with intrapair differences in BMI, at least among men, in contrast to the findings of the present study. In another study [38], the association between genetic predisposition to high BMI (as estimated on the basis of 32 BMI-associated loci) and SSBs was higher among participants with higher intake of such beverages than among those with lower intake. In yet another study, soft drinks were associated with a higher body weight gain among participants in three Danish cohorts [39]. Moreover, the authors reported that a genetic predisposition to a high waist circumference may attenuate the association between soft drink consumption and body weight gain, whereas a genetic predisposition to high BMI and overall adiposity strengthened the association between soft drink intake and abdominal fat gain. Together, the results of the studies noted above and the present study are consistent and demonstrate that investigators examining associations between soda consumption and BMI should carefully consider additional variables, including genetic factors and/or shared environmental factors that may lead to both more soda drinking and higher BMI.

An important caveat of the present study is that we used BMI as the outcome, and it is well accepted that BMI is a simple anthropometric measure commonly used to classify overweight and obesity status but does not measure body fat or body fat distribution. Among adults from the Framingham Heart Study Offspring and Third Generation Cohorts, SSB consumption was associated with visceral adipose tissue (VAT) volume in a cross-sectional analysis [40] whereas higher SSB intake was associated with greater change in VAT volume prospectively [41]. It is also well accepted that VAT is closely related to metabolic disturbances including insulin resistance. Along these lines, regular SSB intake was associated with a greater increase in insulin resistance and a higher risk of developing prediabetes [42] and fatty liver disease [43] among middle-aged adults in the same Framingham cohorts noted above. Thus, SSBs, including soda, may result in deleterious effects on fat partitioning and cardiometabolic disease risk factors beyond any potential effects on BMI per se.

### Strengths and limitations

The primary strength of this study is its use of twin pairs as subjects, which provides a unique opportunity to control for genetic and shared environmental effects from rearing on exposures and outcomes of interest. Additionally, its large sample size from a community-based twin registry allows for greater assumed power.

On the other hand, the cross-sectional design of this study limits our ability to infer causality in the soda consumption-BMI relationship because we do not know the temporality of the association. Thus, our conclusions are limited to “quasi-causal” effects. Additionally, the structure of data collection provides some limitations to the study. Data was self-reported, and both dietary patterns and body weight are subject to self-report bias. Furthermore, there was no differentiation between diet and non-diet soda, and between caffeinated and non-caffeinated soda, thus limiting generalizability of results to specific types of soda. However, soda production in the U.S. is dominated by regular carbonated soft drinks (i.e., non-diet soda) [44], therefore, these results are at least generalizable to most studies of associations between non-diet sodas and BMI. Finally, the racial makeup of the population was largely homogenous, limiting the generalizability of the results to populations that differ in terms of race/ethnicity and socioeconomic status.

## Conclusions

The significant association between soda consumption and BMI observed among all twins (the phenotypic association) was greatly reduced and no longer significant within twin pairs, and the lack of association within pairs was due to genetic confounding. This suggests that the association between soda consumption and BMI commonly reported in many studies may be mediated by genetic factors that are common to both soda drinking and BMI.
